# Chitosan-Functionalized Graphene Nanocomposite Films: Interfacial Interplay and Biological Activity

**DOI:** 10.3390/ma13040998

**Published:** 2020-02-23

**Authors:** Natalia Wrońska, Aicha Anouar, Mounir El Achaby, Katarzyna Zawadzka, Marta Kędzierska, Katarzyna Miłowska, Nadia Katir, Khalid Draoui, Sylwia Różalska, Ireneusz Piwoński, Maria Bryszewska, Abdelkrim El Kadib, Katarzyna Lisowska

**Affiliations:** 1 Department of Industrial Microbiology and Biotechnology, Faculty of Biology and Environmental Protection, University of Lodz, 12/16 Banacha Street, 90-236 Lodz, Poland; natalia.wronska@biol.uni.lodz.pl (N.W.); katarzyna.zawadzka@biol.uni.lodz.pl (K.Z.); sylwia.rozalska@biol.uni.lodz.pl (S.R.); 2 Euromed Research Center, Engineering Division, Euro-Med University of Fes (UEMF), Route de Meknes, Rond-point de Bensouda, Fès 30070, Morocco; a.anouar@ueuromed.org (A.A.); n.katir@ueuromed.org (N.K.); 3 Materials and Interfacial Systems Laboratory (MSI), Faculty of Sciences, Abdel Malek Essaadi University, B.P. 2121, M’hannech II, Tetouan 930000, Morocco; khdraoui@yahoo.fr; 4 Materials Science and Nano-engineering (MSN) Department, Mohammed VI Polytechnic University (UM6P), Lot 660–Hay Moulay Rachid, Benguerir 43150, Morocco; Mounir.ELACHABY@um6p.ma; 5 Department of General Biophysics, Faculty of Biology and Environmental Protection, University of Lodz, 141/143 Pomorska Street, 90-236 Lodz, Poland; marta.kedzierska@unilodz.eu (M.K.); katarzyna.milowska@biol.uni.lodz.pl (K.M.); maria.bryszewska@biol.uni.lodz.pl (M.B.); 6 Department of Materials Technology and Chemistry, Faculty of Chemistry, University of Lodz, 163 Pomorska Street, 90-236 Lodz, Poland; ireneusz.piwonski@chemia.uni.lodz.pl

**Keywords:** chitosan, graphene, nanocomposites, interfacial assembly, mechanical, biological properties

## Abstract

Graphene oxide (**GO**) has recently captured tremendous attention, but only few functionalized graphene derivatives were used as fillers, and insightful studies dealing with the thermal, mechanical, and biological effects of graphene surface functionalization are currently missing in the literature. Herein, reduced graphene oxide (**rGO**), phosphorylated graphene oxide (**PGO**), and trimethylsilylated graphene oxide (**SiMe_3_GO**) were prepared by the post-modification of **GO**. The electrostatic interactions of these fillers with chitosan afforded colloidal solutions that provide, after water evaporation, transparent and flexible chitosan-modified graphene films. All reinforced chitosan–graphene films displayed improved mechanical, thermal, and antibacterial (*S. aureus*, *E. coli*) properties compared to native chitosan films. Hemolysis, intracellular catalase activity, and hemoglobin oxidation were also observed for these materials. This study shows that graphene functionalization provides a handle for tuning the properties of graphene-reinforced nanocomposite films and customizing their functionalities.

## 1. Introduction

One major line of research in the bio-based polymer industry lies in processing and manufacturing these materials as bioplastics for food preservation and as medical devices, including antimicrobial reagents [1]. Commonly used synthetic packaging materials are enriched with persistent, slowly degradable petroleum-based polymers that generate a considerable amount of waste [2]. Bio-based polysaccharide composites could be excellent alternatives to traditional packaging [3].

Chitosan is an aminocarbohydrate obtained from the incomplete deacetylation of chitin, which is extracted from exoskeletons of crustaceans and is also a primary component of the cell wall in fungi [4]. The presence of amino groups in the chitosan backbone imparts it with catalytic activity [5], metal chelating ability [6], and biological efficiency [4]. Chitosan is moreover biocompatible, fully degradable, water soluble, and can be used as a colloidal solution, triggered as a pH-responsive hydrogel, casted as films, and shaped as self-standing microspheres [7].

The excellent film-forming ability of chitosan has opened great opportunities for bio-based packaging materials [8]. However, the poor mechanical strength of chitosan constitutes a serious impediment for this specific application. The addition of a low amount of nanosized fillers, e.g., montmorillonite [9], graphene oxide [10], hydroxyapatite [11,12,13], carbon nanotubes [14], and zinc oxide nanoparticles [15] provides a way to tune chitosan’s mechanical properties, thermal stability, and antimicrobial activity. Increasing interest has been recently devoted to graphene derivatives for manufacturing advanced functional nanocomposites. Besides, graphene brings additional properties (electronic mobility, conductivity, path tortuosity, catalytic and adsorptive ability, sensing, and biological activity) that open new avenues in biomedicine and wearable electronics. The most useful nanocomposites should have good antimicrobial activity with low cytotoxicity. Some of the studies have indicated that graphene can be toxic to blood cells [16,17]. The hemotoxicity of nanoparticles depends on the size of the nanoparticles used and their oxygen content. On the other hand, pristine and functionalized graphene exhibit a very high hemocompatibility [18]. Therefore, determining the cytotoxic activity of this materials is an important stage of research.

The chemical oxidation of graphite stands as the most practical route to graphene oxide (**GO**) [19]. **GO** can be moreover reduced to **rGO** to restore to some extent the original properties of graphene. As metal-free nanomaterials, graphite, graphene oxide, and graphene were recently explored for biomedical applications [20]. Their biological response varies, depending on their size, dispersion, and surface chemistry, including their carbon-to-oxygen ratio [21]. Some reports have paralleled the variation of both physical properties and biological response to the oxidation state of the graphene surface and indicated the pivotal role of surface chemistry in these nanomaterials [21].

**Figure 1:**
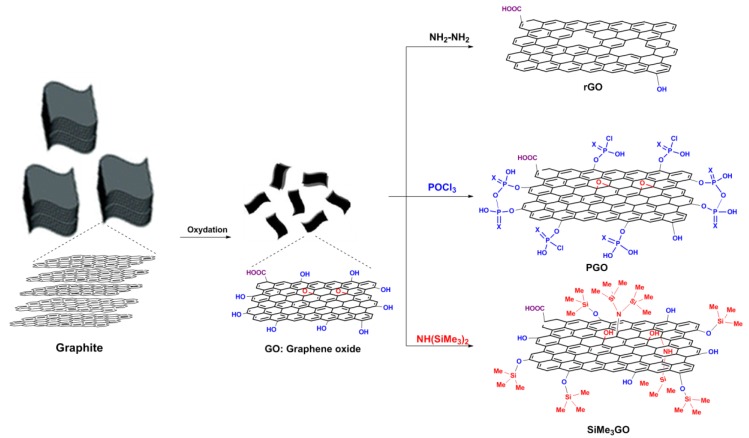
Chemical pathway illustrating the preparation of four fillers (graphene oxide (**GO**), reduced GO (**rGO**), phosphorylated GO (**PGO**), and trimethylsilylated GO (**SiMe_3_GO**) from graphite.

Recently, previous reports have disclosed the synthesis of chitosan–graphene nanocomposites [22,23,24,25], with a special emphasis on their thermal, mechanical, and biological properties [26,27,28]. These reports have clearly substantiated the pivotal role of graphene in these bio-based materials. Considering the importance of thermal, mechanical, and biological properties in active packaging films and biomedical devices, the correlation of these properties, as a function of the filler surface, can be of great interest. Herein, we report the preparation of phosphorus and silicon containing graphene oxide and their comparison with the starting graphene oxide and reduced graphene oxide (Figure 1). These fillers were used to build chitosan-exfoliated-graphene films. Their thermal, mechanical, and biological properties were evaluated and compared to non-modified chitosan and standard chitosan–graphene oxide films to unveil the possible role of graphene surface functionalization in the conceived nanocomposites. We assumed that our research would provide us with an answer to the questions of whether reinforced chitosan–graphene films can improve the thermal and mechanical properties of chitosan as well as enhance the antibacterial activity with low hemotoxicity.

## 2. Materials and Methods

### 2.1. Materials

Commercially available reagents and solvents were purchased from Across and Sigma-Aldrich (St. Louis, MO, USA). Chitosan of medium molecular weight and 85% deacetylation degree was purchased from Sigma-Aldrich (Hamburg, Germany). Graphite flakes, potassium permanganate, sodium nitrate, sulfuric acid, hydrochloric acid, hydrazine, hydrogen peroxide, phosphoryl chloride, bis-trimethylsilylamine, ethanol, tetrahydrofuran, and acetic acid were purchased from Across and Sigma-Aldrich. Phosphate-buffered saline (PBS) was purchased from BioShop (Burlington, ON, Canada). Glutaraldehyde 25% and osmium tetroxide 4% solution were purchased from Agar Scientific (Stansted, UK). Absolute ethanol was purchased from EMSURE (Darmstadt, Germany).

### 2.2. Characterization

Fourier transform infrared (FTIR) spectra were obtained with a Perkin-Elmer Spectrum 100FT-IR spectrometer on neat samples (ATR FT-IR) (resolution of 4 cm^−1^ with 32 scan, PerkinEmler, Shelton, CT, USA). ^13^C and ^31^P CP MAS NMR spectra were acquired on a Bruker Avance 400 WB spectrometer (Bruker Biospin, Rheinstetten, Germany) at 100 MHz and 162 MHz respectively under cross-polarization conditions. Diffuse reflectance UV-visible spectroscopy (DRUV) was measured in the 200–800 nm range using spectral on as the reference on a Perkin-Elmer Lambda 1050 spectrometer equipped with an integrating sphere (PerkinEmler, Labsphere, North Sutton, NH, USA). XPS measurements were conducted on a Versa Probe-II tool from ULVAC-Phi (Chigisaki, Kanagawa, Japan) using a focused monochromated Al Ka radiation (1486.6 eV). Raman spectra were recorded in the backscattering geometry using an In-Via Renishaw Raman spectrometer (532 nm) (Renishaw, Charfield, GL, UK). Ultra-sonication was performed using a VWR (Ultrasonic cleaner) USC-THD (Power 9) (VWR International GmbH, Wien, Austria). The time needed to disperse the two fillers is 180 min. Contact angle measurements were recorded using a dynamic contact angle meter (KRUSS GmbH Easy Drop, Kruss GmbH, Hamburg, Germany) equipped with a charge-coupled device camera and using an image capture program employing scat software (VCA Optima, AST Products, Billerica, MA, USA. The cut film (3 cm × 3 cm) was fixed on the top of a dynamic support. A droplet (3 μL) was placed on the film surface and the change of contact angles was treated by the software (VCA Optima, AST Products, Billerica, MA, USA) of the machine. Each measurement was repeated four times, and their average was considered. Thermogravimetric analysis (TGA) was performed on a Q500 (TA Instruments, New Castle, DE, USA) using a heating rate of 10 °C/min from room temperature to 700 °C under air (Figure S5, ESI). Differential scanning calorimetry (DSC) analyses were carried out on a Q500 (TA Instruments, New Castle, DE, USA) using a heating rate of 10 °C/min from −50 °C to 400 °C under air. Scanning electron microscopy (SEM) images were acquired using a JEOL JSM 6300 apparatus (SEMTech Solution, North Billerica, MA, USA), applying a voltage of 1 kV. Tensile tests were performed using an mpk-LUDWIG UG instrument (LUDWIGmpk, Nordhorn, Germany). The specimens were cut in a rectangular shape with the following dimensions (80 mm in length and 10 mm in width). The results were averaged based on five repeated analysis tests.

### 2.3. Preparation of Modified Graphene Fillers

Graphene oxide (**GO**) was obtained from graphite flakes using the Hummers method [19]. In a typical procedure, graphite flakes (5 g) and NaNO_3_ (2.5 g) were mixed in 150 mL of H_2_SO_4_ (98%) in a 1000 ml volumetric flask kept under at ice bath (0 °C) with continuous stirring. The workup procedure can be found in the supporting information.

**PGO** was obtained through the phosphorylation of graphene oxide using POCl_3_ as the phosphorus source. The details can be found in the supporting information.

**rGO** was obtained following this protocol: hydrazine (0.3 mL) was added to a dispersion of **GO** (16 mg) in 40 mL H_2_O. The mixture was heated at 60 °C for 24 h. Then, the solution was subjected to filtration and extensive washing, followed by precipitate with ethanol. The harvested **rGO** material was finally dried at 60 °C for 12 h.

Silylated graphene oxide (**Me_3_SiGO**) was obtained following this protocol. Bis-trimethylsilylamine (76 mmol) was added to a suspension of **GO** (40 mg in 100 mL of toluene). The mixture was magnetically stirred for 24 h at 80 °C. The powder was recovered by filtration and washed with toluene and dried for 6 h in an oven at 60 °C.

### 2.4. Preparation of Chitosan–Graphene Films

The procedure used to prepare **CS-GO-*f***, **CS-PGO-*f***, **CS-rGO-*f*** or **CS-SiMe3GO-*f*** films is similar to a previous study [10]. At first glance, 50 mg of chitosan was completely dissolved in 4 mL of 1% (*v*/*v*) acetic acid solution, and the mixture was kept under vigorous stirring for 120 min. The modified graphene filler (1.5 mg) was dispersed in 2 mL of the 1% (*v*/*v*) acetic acid solution and was subjected to sonication for 90 min (except for **rGO** and **SiMe3GO**, where longer sonication times were required). The amount of the filler corresponds to 3 wt % with respect to the biopolymer. The filler suspension was gradually added to the chitosan solution, and the resulting mixture was stirred for an additional 90 min. The resulting solution was cast into plastic Petri dishes allowing solvent removal and film formation after complete drying.

### 2.5. Determination of Antimicrobial Activity

The antimicrobial activity of modified chitosan films against *Staphylococcus aureus* (ATCC 6538) and *Escherichia coli* (ATCC 25922) was evaluated using the Japanese Industrial Standards JIS Z 2801:2000 (https://infostore.saiglobal.com/en-us/Standards/JIS-Z-2801-2000-634364_SAIG_JSA_JSA_1462706/) with modification.

Gram-positive bacteria of *S. aureus* or Gram-negative *E. coli* were cultured on Luria Bertani (LB) medium at 37 °C on a rotary shaker. After the incubation, the test inoculum of *S. aureus* or *E. coli* containing 1 × 10^5^ colony-forming units (CFU per mL) in 500-fold diluted LB-medium was prepared. Next, the bacterial suspension was transferred to chitosan films of 2 cm × 2 cm. Native chitosan films were examined as control samples. After dripping the suspension of *S. aureus* or *E. coli* on the films, each sample was covered with a sterile film (1.7 × 1.7 cm). The samples were incubated in the moist chamber in the dark for 24 h at 37 °C. Next, they were put in aseptic Falcon tubes containing phosphate buffer, vortexed, and removed from the Falcon tubes. A serial dilution was performed with the remaining solution in the phosphate buffer. Out of each dilution, 100 µL of bacterial suspension was seeded on an agar plate and incubated for 24 h at 37 °C. After incubation, viable cells of tested bacteria were counted.

Each type of tested film was examined in triplicate and analyzed individually in four independent experiments. The antimicrobial activity of the tested films was calculated as the percentage of bacterial growth inhibition (SD) toward control films without graphene compounds.

### 2.6. Permeability of Bacterial Cell Membranes

Bacterial suspensions acquired after incubation on chitosan films were washed twice with phosphate-buffered saline (0.1 M, pH = 7.4) and incubated with 3 µM of propidium iodide in the darkness, for 15 min, at room temperature. Then, the cells were washed twice with PBS, and 10 µL each suspension was mounted on a microscopic slide.

#### Confocal Microscopy

The images were done using a CLSM confocal laser scanning microscope (LSM 510 Meta, Zeiss, Jena, Germany) with an Axiovert 200 M (Zeiss, Jena, Germany) and a Plan-Apochromat objective (100×/1.4 Oil DIC). The propidium iodide fluorescence was detected at laser 543 nm and 560–615 nm, and the Nomarski DIC sections were done at the same laser line. All figures in this paper are representative samples based on observation.

### 2.7. Morphological Changes of *S. Aureus* Cells Visualized by Scanning Electron Microscopy (SEM)

*S. aureus* cells treated with **CS-GO-*f*** and **CS-PGO-f** were washed with phosphate-buffered saline (PBS) and vortexed for 3 min. Next, cells were washed three times with PBS and centrifuged at 10,000 rpm for 5 min. Bacterial cells were suspended in a solution of glutaraldehyde and incubated for 20 h. Next, bacterial cells were centrifuged at 10,000 rpm for 5 min and washed three times with PBS. Fixed cells were suspended in osmium tetroxide solution and incubated for 20 min. Subsequently, bacterial cells were centrifuged and washed three times in PBS and dehydrated in ethanol solutions (25%, 50%, 75%, 90%, and 100%) for 10 min each. The cells were spread on a silicon wafer, dried at 22 °C, and sputtered with a gold layer at 2 nm thickness. SEM images of *S. aureus* cells were visualized using a scanning electron microscope–Nova NanoSEM 450 (FEI, Hillsboro, OR, USA). SEM analyses were performed in an immersion mode with using a through-lens detector (TLD) at a magnification of 80,000×.

### 2.8. Hemolysis Assay

Blood from healthy donors was obtained from the Regional Blood Donation and Blood Treatment Center in Lodz, Poland. Erythrocytes were isolated from blood by centrifugation (3000 rpm, 10 min) at 4 °C, washed three times with PBS (phosphate-buffered saline; pH = 7.4) and used immediately after separation. To study the impact of graphene composites on red blood cells (RBCs), washed erythrocytes (hematocrit, HTC 2%) were treated with films in the form of squares (0.5 × 0.5 cm). RBCs suspended in PBS (without graphene composite) were used as a control. The samples were incubated at 37 °C for 1, 3, and 24 h. Next, samples were centrifuged at 3000 rpm for 10 min, and the absorbance of the supernatant was measured spectrophotometrically at 540 nm (Jasco V-650, Jasco International Co., Osaka, Japan). The percentage of hemolysis was determined based on the hemoglobin (Hb) amount released into the supernatants and calculated using the following formula: $\%\;\mathrm{Haemolysis}\;=A_{s}/A_{c}\;\times \;100\%$ where *A_s_* is the absorbance of the sample and *A_c_* is the absorbance of the samples containing erythrocytes in water (100% of hemolysis).

### 2.9. The Adsorption of Hemoglobin (Hb)

The adsorption of hemoglobin onto graphene composite was also investigated. Graphene composite squares (0.5 cm × 0.5 cm) were added to hemoglobin solutions (0.1% *v*/*v*) and were incubated at 37 °C for 3 or 24 h. Next, the absorbance of the hemoglobin solution was measured at 540 nm. The percentage of hemoglobin adsorption was calculated from the formula: $\mathrm{Adsorption}\;\mathrm{of}\;\mathrm{Hb}\;=\;100\%-\left(A_{s}/A_{c}\;\times \;100\%\right)$, where *A_s_* is the absorbance of the sample containing the graphene composites, and *A_c_* is the absorbance of the control without graphene composites.

### 2.10. Methemoglobin (Met-Hb)

Methemoglobin was determined spectrophotometrically based on the absorption spectrum in the range from 440 to 700 nm (Jasco V-650). The percentage of met-Hb in the sample was calculated from the absorbance at 630 and 700 nm. Hemoglobin treated with potassium ferricyanide (100% met-Hb) was used as a positive control. $\%\;\mathrm{of}\;\mathrm{met}-\mathrm{Hb}\;=\;\left(A_{630}-A_{700}\right)/\left(A_{630}^{*}-A_{700}^{*}\right)\;100\%$ where: *A*_630_ = the absorbance of a sample with/without graphene composites at 630 nm, *A*_700_ = the absorbance of a sample with/without graphene composites at 700 nm, A^*^_630_ = the absorbance of a sample with/without graphene composites treated with potassium ferricyanide (100% met-Hb) at 630 nm, and A^*^_700_ = the absorbance of a sample with/without graphene composites treated with potassium ferricyanide (100% met-Hb) at 700 nm.

### 2.11. Catalase Activity

The catalase (CAT) activity in erythrocytes was determined by the method of Aebi [29]. Erythrocytes were incubated for 3 or 24 h at 37 °C with/without graphene composite squares (0.5 cm × 0.5 cm). The enzyme activity was determined in hemolysates in the presence of 0.06% H_2_O_2_ diluted in 50 mM phosphate buffer (pH = 7.0). The reaction was carried out for 1 min, measuring absorbance at λ = 240 nm with a Jasco V–650 spectrophotometer. One unit of catalase activity was defined as the activity required to degrade 1 µmol of hydrogen peroxide in 60 s. Catalase activity was calculated in relation to mg of hemoglobin in hemolysates. The calculation was based on the following formula:

(1) Activity (U/mL)=(ΔA∗R)/0.0145

where: ΔA = a decrease in absorbance tested at λ = 240 nm, R = the sample dilution, and 0.0145 = the micromolar absorption coefficient for hydrogen peroxide. The concentration of hemoglobin in the hemolysates was measured by the method of Drabkin [30]. The results are presented as a percentage of the control.

### 2.12. Statistical Analysis

Data are presented as mean ± SD from six sets of measurements. The statistical differences between the control and treatment groups and differences between films were analyzed by one-way ANOVA followed by Tuckey’s analysis. *p* < 0.05 was accepted as being statistically significant.

## 3. Results

### 3.1. Synthesis of Functionalized Graphene Fillers

The preparation of the fillers used in this study is illustrated in Figure 1. Graphene oxide (**GO**) was prepared via the chemical oxidation of graphite followed by ultra-sonication to afford highly dispersed sheets [19]. Subjecting **GO** to phosphoryl trichloride in THF, in the presence of K_2_CO_3_ as a base, resulted in tethering its surface with phosphorus motifs, giving rise to **PGO** material as previously described [31]. **GO** was also subjected to hydrazine treatment to remove the remaining oxygen functional groups [32]. The reduced graphene oxide (**rGO)** displays fewer oxygen groups on its surface compared to the starting **GO** precursor. We also undertook a gentle silylation of **GO** using hexamethyldisilazane (HMDS). The functionalization of this filler resulted in the introduction of trimethylsilyl groups in its surface to provide **SiMe_3_GO** (Figure 1).

More information about the synthesis of functionalized graphene fillers are detailed in the supporting information (Figures S1–S4, ESI).

### 3.2. Preparation and Characterization of Chitosan-Modified Graphene Films

**Figure 2:**
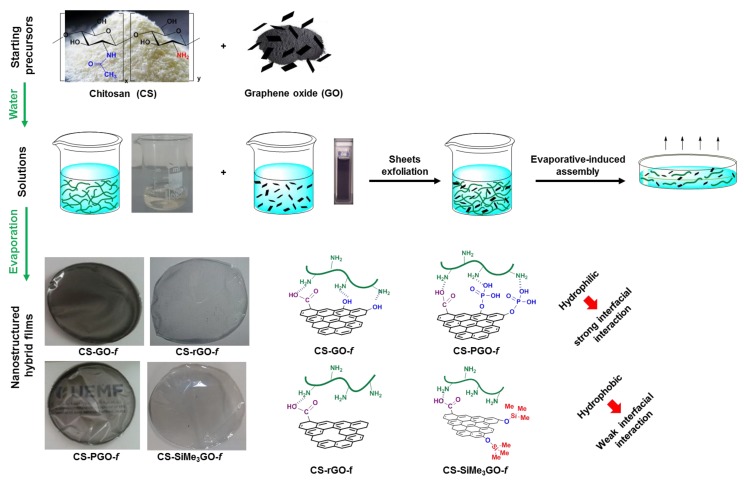
Preparation of chitosan-reinforced-functionalized graphene films. From top to bottom: raw precursors, their solutions, and their subsequent evaporation to provide transparent films. Digital photos of the four films as prepared. Right. Illustration of the molecular interplay occurring at the nanocomposite interface.

With these fillers in hand, we next set out to build four different chitosan–graphene films. Evaporation-induced assembly of the aqueous colloidal solution (chitosan and 3 wt % modified graphene in water) afforded transparent and flexible crack-free films. For comparison, non-reinforced chitosan film was also prepared by casting the colloidal solution of soluble chitosan carbohydrate. Interestingly, irrespective of the filler used, the as-prepared films are more transparent than the one reinforced with **GO** (Figure 2).

**Figure 3:**
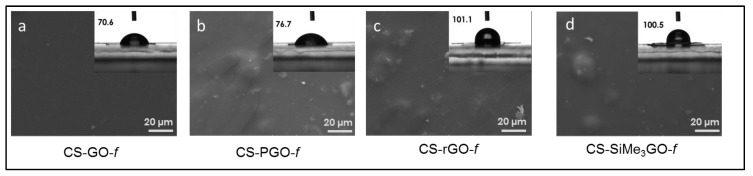
SEM analyses and contact angle measurement (onset) of: **CS-GO-*f*** (**a**), **CS-PGO-*f*** (**b**), **CS-rGO-*f*** (**c**), and **CS-SiMe3GO*-f*** (**d**).

FTIR analysis was undertaken to gain insight into the interplay occurring between nitrogen-containing groups of chitosan and the modified graphene filler (Figure S6, ESI). All the nanocomposite films reveal the signature of chitosan biopolymer. **CS-PGO-*f*** displays some slight changes where the peaks corresponding to carbonyl stretching in NHCOCH_3_ and NH_2_ bending are shifted to higher values (from 1638 cm^−1^ and 1549 cm^−1^ for neat chitosan to 1645 cm^−1^ and 1556 cm^−1^, respectively). This suggests the occurrence of strong interfacial interactions with the phosphonic groups of the filler [33]. The bands corresponding to NH_2_ twisting and C-OH stretching are intensified in **CS-PGO-*f*** nanocomposites [23]. The FTIR spectrum of **CS-SiMe_3_GO-*f*** displays new bands at 1582 cm^−1^ characteristic of the C=C bonds present in **GO**. An additional peak at 858 cm^−1^ can be found in the spectrum characteristic of Si-C bonds, which might be due to the presence of residual Si-CH_3_ [34]. SEM (scanning electron microscopy) was conducted to provide information about the surface of these films (Figure 3). A smooth surface can be seen in the case of **CS-GO-*f***, whereas some aggregates can be seen in the case of **CS-PGO-*f***, **CS-SiMe_3_GO-*f***, and **CS**-**rGO-*f***. Contact angle measurements were conducted to assess the wettability of the reinforced nanocomposites (Figure 3, Table S1, ESI), and they are as follows: **CS-*f*** (73.3° ± 2.18), **CS-GO-*f*** (70.6° ± 1.22), **CS-PGO-*f*** (76.7° ± 1.14), **CS-rGO-*f*** (101.1° ± 3.67), and **CS-SiMe_3_GO-*f*** (100.5° ± 2.27). In the case of **CS-SiMe_3_GO-*f*** and **CS-rGO-*f***, an increase in their contact angle value points to a significant variation in their surface wettability with respect to the non-modified chitosan film, in super consistency with their surface hydrophobization. **PGO** provides a similar contact angle value as for native chitosan films, while a slight decrease was found upon the use of **GO**. This discrepancy can be explained by several factors: (i) the higher wettability for **CS-GO-*f*** than that of chitosan due to a better dispersion of this filler within chitosan probably due to high interactions between the oxygen-rich surface of **GO** and amino groups of **CS** [35]; (ii) the presence of additional oxygenated groups on **CS-GO-*f*** that emanate from the graphene oxide surface; and (iii) lastly, the smooth surface in **CS-GO-*f*** compared to its roughness for the other analogue films.

**Figure 4:**
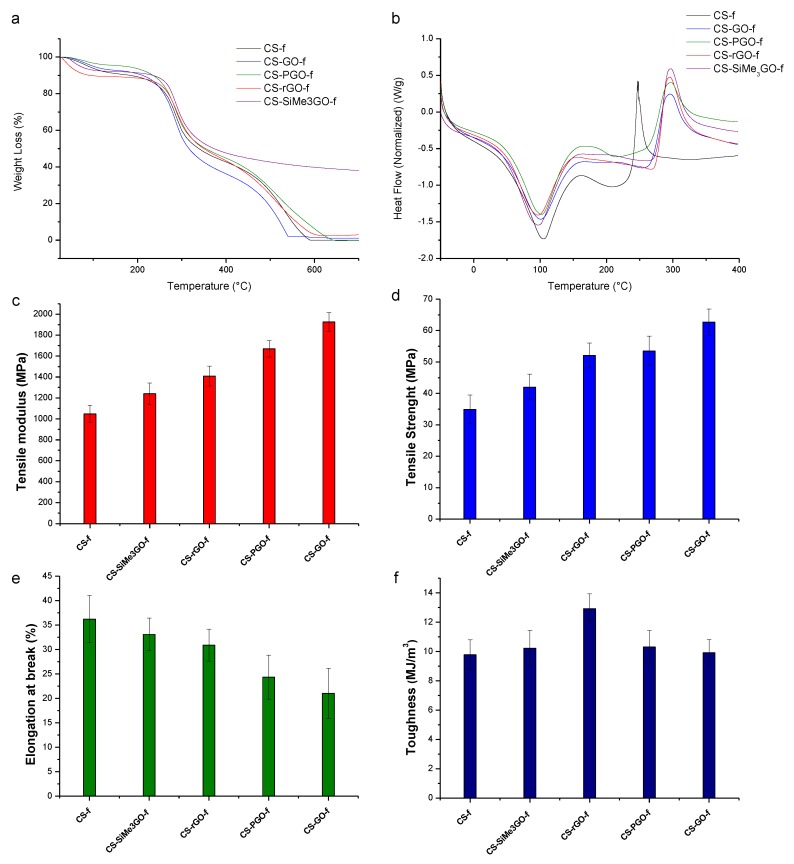
Thermal and mechanical properties of the resulting nanocomposites. (**a**) Thermogravimetric analysis (TGA), (**b**) Differential scanning calorimetry (DSC), (**c**) Tensile modulus, (**d**) Tensile strength, (**e**) Elongation at break, and (**f**) Toughness.

The thermal stability of these films was next investigated using thermogravimetric analysis under air. The weight loss profile showed a diverging pattern depending on the nature of the filler (Figure 4a). The most remarkable trend was related to the film reinforced with silylated **SiMe_3_GO** filler, which afford 38 wt % char residues at 700 °C. In contrast, total decomposition occurs with no char residue for native **CS-*f***, **CS-GO-*f***, **CS-rGO-*f***, and even with **CS-PGO-*f***. Within the nanocomposite series, **CS-GO-*f*** shows the fastest degradation profile among the four films, owing to the vulnerability of **GO** and its own oxidative catalytic ability [10,36].

The thermal behavior of these nanocomposite films was further evaluated by DSC analysis under nitrogen (Figure 4b). Two main peaks were observed for all the samples studied. The initial endothermic peak observed at approximately 90 °C is due to the evaporation of water molecules contained in the films, forming intermolecular hydrogen bonds via the free hydroxyl and amine groups of chitosan. The second sharp exothermic peak was observed at approximately 300 °C and is due to the degradation of chitosan units [37]. Notably, a significant shift of this value was observed when comparing native chitosan films (T = 247 °C) and the reinforced chitosan-modified graphene films (T = 296 °C).

Figure 4c–f show the tensile modulus, tensile strength, elongation at break, and toughness of all the studied samples. The typical stress–strain curves of chitosan and chitosan-modified graphene nanocomposites are in the supplementary information, Figure S7, ESI). The use of 3 wt % of the filler enhanced the mechanical properties of the resulting nanocomposites. **CS-GO-*f*** displays the highest tensile modulus, which is consistent with previous reports [10,38]. This enhancement was attributed to the easy dispersion of **GO** reached, owing to the strong hydrogen bonding between chitosan and the oxygenated filler. Although the films reinforced with **CS-SiMe_3_GO-*f*** display the lowest tensile strength, this nanocomposite maintains, to some extent, a good flexibiliy with an elongation at break of 33% versus 36% for neat chitosan films. The reduction of **GO** (to **rGO**) affects the dispersion of the sheets and provides only a few interacting oxygenated sites in **CS-rGO-*f*** in comparison to **CS-GO-*f*** accounting for the low tensile strength found. **CS-PGO-*f*** displays a lower tensile strength than **CS-GO-*f,*** which could be explained by a slight worsening of the dispersion of **PGO** within the matrix. This is mainly due to the aggregation of **PGO** at the solid state, as it can be evidenced by the shift of the (002) peak to higher 2θ values in XRD [31]. However, stiff materials were obtained by **GO** loading, whereas the elongation at break observed for **CS-PGO-*f*** implies that the stretchability of this material is kept. To summarize, a good dispersion of these fillers as well as an optimal loading have to be achieved in order to tune the properties of these materials.

### 3.3. Biological Activity of Chitosan-Modified Graphene Films

#### 3.3.1. Antimicrobial Activity

**Figure 5:**
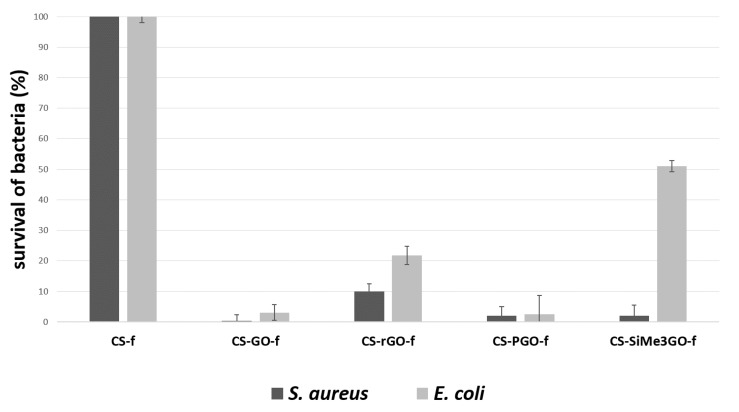
Antibacterial activity of the resulting nanocomposite films; inhibition (%) of *S. aureus* and *E. coli* after 24 h incubation.

The antimicrobial activity of chitosan-reinforced graphene films (**CS-GO-*f***, **CS-rGO-*f***, **CS-PGO-*f***, and **CS-SiMe_3_GO-*f***) was assessed using *Staphylococcus aureus* ATCC 6538 and *Escherichia coli* ATCC 25922 as model bacteria and taking native chitosan film **CS-*f*** as a reference (Figure 5). All chitosan-reinforced graphene nanocomposites caused a high inhibition of *S. aureus* growth compared to the native chitosan film. In the case of **CS-GO-*f***, 100% inhibition of *S. aureus* growth was observed.

The other samples also showed great antibacterial activity, inhibiting the growth of Gram-positive strain by 90–95%. Satisfactory results were also obtained for *E. coli*. In **CS-GO-*f*, CS-rGO-*f*, CS-PGO-*f***, and **CS-SiMe_3_PGO-*f***, the growth of *E. coli* was inhibited by 90–95%. These results were similar to the results for *S. aureus*. The weaker effect was observed for the most hydrophobic material, **CS-SiMe_3_GO-*f***, where bacterial growth was inhibited by only 50%, suggesting the importance of the surface wettability for bacterial adhesion and indicating that the mechanism of the bacterial growth involves surface adsorption. Furthermore, a previous study demonstrated the importance of wettability of silica, where it was found that high concentrations of hydrophobic silica were crucial to impart these materials with high antimicrobial activity [39]. Previously, Mazaheri et al. [40] reported the antibacterial activity of **CS-GO** composites against *S. aureus*. The results showed more than 77% cell inactivation after 3 h of incubation. The great inhibition of *S. aureus* and *E. coli* was also recorded by chitosan–graphene oxide–polyhexamethylene guanidine hydrochloride composites, providing 92% and 95% growth inhibition, respectively [41]. Significant antibacterial activity against methicillin-resistant *S. aureus*, *S. aureus*, *E. coli*, and the opportunistic dermal pathogen *C. albicans* was also exhibited by chitosan–iron oxide-coated graphene hydrogel films [42].

In order to confirm that bacterial cell membranes were disrupted, we treated samples of **CS-GO*-f***, **CS-PGO-*f***, and **CS-SiMe_3_PGO-*f*** (which had the best antibacterial properties) with propidium iodide (PI). Propidium iodide does not cross the intact membrane of bacterial cells due to the retained electric charge. Confocal microscopy and propidium iodide staining of *S. aureus* cells after incubation on the pure and chitosan-modified graphene films was performed. The results confirm the strong antibacterial properties of the samples tested, because all microorganisms per microscope field were stained red by PI, excluding the control (Figure S8, Table S2, ESI).

**Figure 6:**
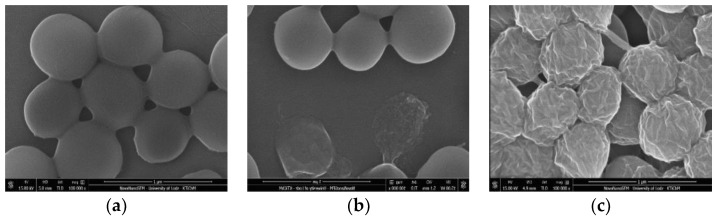
SEM images of *S. aureus* incubated with or without **CS-GO-*f*** and **CS-PGO-*f.*** (**a**) untreated, (**b**) treated of **CS-GO-*f****,* and (**c**) treated of **CS-PGO-*f***.

It is most likely that the antimicrobial mechanism of graphene-based chitosan nanocomposites may be caused by the direct contact and interaction of the graphene sharp nanosheets with the bacterial cell membranes, resulting in an alteration of membrane permeability. Then, these changes cause cellular deformation and surface perforation [43,44]. The results confirm that chitosan films can affect the permeability of *S. aureus* cell membranes. Clarification of the mechanism of action and show the changes in the morphology of *S. aureus* after treatment with **CS-GO-*f*** (Figure 6b) and **CS-PGO-*f*** (Figure 6c) films was sought using scanning electron microscopy (SEM). The control sample was the untreated bacterial cells (Figure 6a). The SEM images of *S. aureus* treated with chitosan-modified films indicated that the cell wall membranes are the essential sites of action. After 24 h of incubation with chitosan-reinforced graphene films, we observed a leakage of cellular components or cells with shrunken appearance compared to the untreated control. This result is correlated with the permeability data determined on a confocal microscope (Figure S8, Table S2, ESI). We observed the largest changes in *S. aureus* morphology after treatment with **CS-PGO-*f*** (Figure 6c). The highest permeability (98%) of *S. aureus* cell membrane was also determined for the same sample. In summary, various mechanisms of antibacterial activity of graphene materials have been suggested such as membrane stress, oxidative stress, and electron transfer [45]. However, destruction of the bacterial cell membranes is suggested as an essential mechanism of antibacterial action by graphene materials [46,47].

#### 3.3.2. Hemolysis

**Figure 7:**
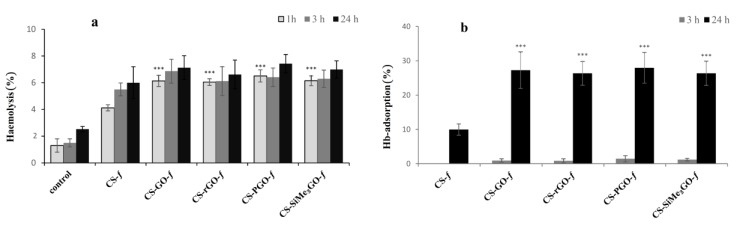
Hemolysis (**a**) and hemoglobin adsorption (**b**) of chitosan-reinforced graphene films.

Figure 7a shows the hemolytic activity after incubation times of 1, 3, and 24 h. All chitosan-reinforced graphene nanocomposite films induced hemolysis. After incubation for 1 and 3 h, the hemolysis of erythrocytes was approximately 6.5% and after 24 h incubation, the hemolysis increased to 7–7.5%, but all these changes are not statistically significant. As hemolysis was not dependent on incubation time, we investigated possible hemoglobin adsorption on the surface of chitosan-reinforced graphene films. After 3 h incubation of hemolysate with graphene composites, a negligible adsorption of hemoglobin was experienced. However, hemoglobin adsorption reached 22–29% after 24 h of incubation (Figure 7b). The lowest adsorption percentage was observed for **CS-SiMe_3_GO-*f***, which was in agreement with its low wettability and the pronounced hydrophobic character of the trimethylsilylated filler. These results suggest that hemoglobin released from erythrocytes remains adsorbed to chitosan–graphene films after 24 h, which causes a decrease in the hemoglobin content in the solution and was misread as a lack of hemolysis increase after 24 h incubation. Thus, the percentage of hemolysis after 24 h does not reflect real hemolytic activity but is rather associated with the accumulation of hemoglobin on the surface of graphene composites. A similar effect was previously observed with functionalized-SBA-15-type mesoporous materials [48]. Significant damage of the erythrocyte membrane by **GO** and graphene sheets was previously reported, leading to a dose-dependent hemolytic activity on RBCs [17]. In the case of dispersed **GO** sheets, the extent of exfoliation and particle size play critical roles in inducing hemolysis. Indeed, sonicated **GO** that decreased in size exhibited higher hemolytic activity than untreated particles that were assumed to be larger. Compared to individually dispersed **GO** sheets having higher surface oxygen content, the aggregated graphene sheets showed lower hemolytic activity. The mechanism of action in suspension is fundamentally different from the mechanism of action occurring in hydrogels or in solid-state films. Indeed, the hemolysis of suspended graphene oxide can be totally prevented by covering the sheets by chitosan [49]. Herein, chitosan–graphene oxide films do not follow a similar trend, and substantial hemolytic activity was observed despite the presence of the chitosan network. Our data allow us to conclude that chitosan–graphene nanocomposites, regardless of surface modification, affect the membrane of blood cells, probably by deformation of the membrane, leading to its damage and the release of hemoglobin. The proteins present on the surface of erythrocytes may also be adsorbed on the surface of chitosan–graphene films, which can have a significant impact on reducing the lifetime of red blood cells [50]. Liao et al. [17] showed that derivatives of graphene oxide (**GO**) interact with neutral, positively, and negatively charged lipid membranes. Disruption of the erythrocyte membrane can be due to electrostatic interactions between **GO** and the positive groups of phosphatidylcholine present in the outer monolayer of erythrocytes membrane. **GO** surface coatings may improve **GO** hemocompatibility.

#### 3.3.3. Intracellular Catalase (CAT) Activity and Hemoglobin Oxidation

We next turned our attention to assess the intracellular catalase activity. CAT is an important antioxidant enzyme that is essential for the organism’s defense against excessive reactive oxygen species (ROS), and its activity variation can be used to reflect the oxidation-reduction equilibrium in cells. Literature data suggest that graphene oxide influences changes in the secondary structure of proteins and their activity [51]. It has been demonstrated that an aqueous solution of graphene oxide (1 mg·mL^−1^) causes a decrease in standard catalase activity [51]. For this reason, we focused on studying the ability of chitosan–graphene bionanocomposite films to trigger the oxidation of hemoglobin in erythrocytes and to affect the activity of intracellular catalase. A decrease in CAT activity is generally associated with an increased content of hydrogen peroxide in the cell [52].

**Figure 8:**
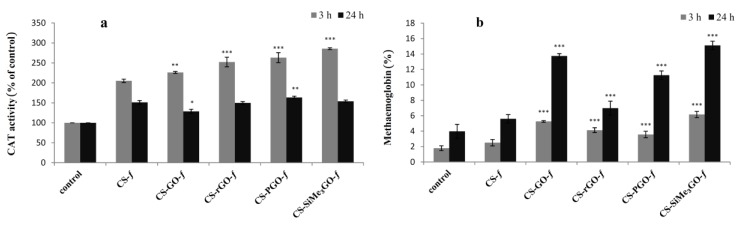
Catalase activity (**a**) and hemoglobin oxidation (**b**) of chitosan-reinforced graphene films.

In our experiments, after 3 h of incubation with chitosan–graphene composite films, the activity of erythrocyte catalase increased significantly compared to that of the control (Figure 8a). After 24 h of incubation, the activity of catalase decreased to 128–167% of the control value. The highest catalase activity observed was for **CS-SiMe_3_GO-*f*** after 3 h of incubation (286%), and the lowest was observed for **CS-GO-*f*** after 24 h of incubation (128%).

It was also shown that an increase in CAT activity is inversely correlated with hemoglobin oxidation [53], and therefore, a decreased activity of this enzyme correlates with the increased methemoglobin (met-Hb) level in RBCs. Thus, we investigated hemoglobin oxidation with these materials. All chitosan–graphene films caused the oxidation of hemoglobin after 3 h of incubation with the erythrocytes (Figure 8b). For the control, the percentage of methemoglobin after 3 h of incubation was only 1.8%, and after 24 h, the percentage increased to 4%. After 3 h incubation with chitosan-reinforced graphene films, the highest methemoglobin content was in the sample incubated with **CS-SiMe_3_GO-*f***. Statistically significant changes in the percentage of met-Hb content were observed for all graphene composites after 24 h of incubation. The highest content of met-Hb was for **CS-SiMe_3_GO-*f*** (15.1%), and the lowest content was recorded for **CS-rGO-*f*** (7.0%).

## 4. Conclusions

We explored the association of chitosan with different surface-functionalized graphene fillers to design four chitosan–graphene films. Whatever the modified graphene filler, chitosan was proven to be effective for exfoliating carbon sheets, thereby yielding flexible and transparent nanocomposite films. The thermal degradation of these films was significantly delayed in the reduced graphene version compared to chitosan–graphene oxide, which clearly demonstrates the detrimental role of residual oxygenated groups on thermal stability of the resulting films. Unexpectedly, high char residue was obtained for films reinforced with a silylated filler, which points to their promising flame-retardant properties. Substantial improvement in the mechanical properties was also achieved in chitosan-reinforced with functionalized graphene films compared to non-modified chitosan films. Notably, graphene incorporation in chitosan films imparts chitosan-reinforced graphene films with potential biological activity. In particular, we demonstrated the highest antibacterial activity of graphene-based chitosan films against Gram-positive (*S. aureus* ATCC 6538) and Gram-negative (*E. coli* ATCC 25922) strains compared to neat chitosan films. Additionally, all tested chitosan–graphene nanocomposites caused erythrocyte hemolysis, adsorbed the released hemoglobin, oxidized hemoglobin, and changed catalase activity. In summary, our results provide information on stable, antibacterial nanocomposites that may help to solve the problems connected with the environmental pollution caused using synthetic packaging materials.

## Supplementary Materials

The following are available online at https://www.mdpi.com/1996-1944/13/4/998/s1, Figure S1: ^13^C Solid-State NMR Spectrum of Graphene Oxide. (**a**) ^13^C Solid State NMR spectrum of graphene oxide; (**b**) ^13^C Solid-state NMR spectrum of **PGO, Figure S2**. Raman spectrum of **GO** and **PGO**. (**a**) Raman spectrum of **GO;** (**b**) Raman spectrum of **PGO**; (**c**) Raman spectrum of **rGO**, Figure S3: XPS Analysis of GO, PGO, and SiMe_3_GO. (**a**) XPS analysis of **GO**; (**b**) XPS analysis of **PGO**; (**c**) XPS analysis of SiMe_3_GO, Figure S4: Water dispersion of functionalized graphene fillers, Figure S5: TGA of **GO**, **PGO**, **rGO**, and **SiMe3GO**, Figure S6: DRIFT analysis of graphene-reinforced chitosan films, Figure S7: Stress–strain curves of chitosan and chitosan-modified graphene nanocomposites, Figure S8: Permeability of *S. aureus* cell membrane after the treatment with chitosan films, Table S1: Contact angle, thermal analysis, and mechanical properties of modified chitosan films, Table S2: PI Permeability (%) of cell membranes of S. *aureus* after incubation with modified chitosan films.
