# Supplementary material for: Chitosan-Functionalized Graphene Nanocomposite Films: Interfacial Interplay and Biological Activity
Source: Materials (Basel). 2020 Feb 23;13(4):998. doi: 10.3390/ma13040998 (PMC7078879; doi:10.3390/ma13040998)
Supplement: Supplementary file 1 [file materials-13-00998-s001.pdf]

materials

MDPI

Supplementary Materials

Chitosan-functionalized Graphene Nanocomposite Films: Interfacial Interplay and Biological Activity

Natalia Wrońska [1], Aicha Anouar [2,3], Mounir El Achaby [4], Katarzyna Zawadzka [1], Marta Kędzierska [5], Katarzyna Miłowska [5], Nadia Katir [2], Khalid Draoui [3], Sylwia Różalska [1], Ireneusz Piwoński [6], Maria Bryszewska [5], Abdelkrim El Kadib [2,*] and Katarzyna Lisowska [1,*]

[1] Department of Industrial Microbiology and Biotechnology, Faculty of Biology and Environmental Protection, University of Lodz, 12/16 Banacha Street, 90-236 Lodz, Poland; natalia.wronska@biol.uni.lodz.pl (N.W.); katarzyna.zawadzka@biol.uni.lodz.pl (K.Z.); sylwia.rozalska@biol.uni.lodz.pl (S.R.)

[2] Euromed Research Center, Engineering Division, Euro-Med University of Fes (UEMF), Route de Meknes, Rond-point de Bensouda, 30070, Fès, Morocco; a.anouar@ueuromed.org (A.A.); n.katir@ueuromed.org (N.K.)

[3] Materials and Interfacial Systems Laboratory (MSI), Faculty of Sciences, Abdel Malek Essaadi University, B.P. 2121, M'hannech II, Tetouan, Morocco; khdraoui@yahoo.fr

[4] Materials Science and Nano-engineering (MSN) Department, Mohammed VI Polytechnic University (UM6P), Lot 660–Hay Moulay Rachid, 43150 Benguerir, Morocco; Mounir.ELACHABY@um6p.ma

[5] Department of General Biophysics, Faculty of Biology and Environmental Protection, University of Lodz, 141/143 Pomorska Street, 90-236 Lodz, Poland; marta.kedzierska@unilodz.eu (M.K.); katarzyna.milowska@biol.uni.lodz.pl (K.M.); maria.bryszewska@biol.uni.lodz.pl (M.B.)

[6] Department of Materials Technology and Chemistry, Faculty of Chemistry, University of Lodz, 163 Pomorska Street, 90-236 Lodz, Poland; ireneusz.piwonski@chemia.uni.lodz.pl

* Correspondence: a.elkadib@ueuromed.org (A.E.K.); katarzyna.lisowska@biol.uni.lodz.pl (K.L.); Tel.: +484-2635-4468

Experimental Section: Preparation of Functional Graphene Fillers

Graphene oxide (**GO**) was obtained from graphite flakes using the Hummers method [19]. In a typical procedure, graphite flakes (5 g) and $NaNO_3$ (2.5 g) are mixed in 150 mL of H_2SO_4 (98%) in a 1000 ml volumetric flask kept under at ice bath (0 °C) with continuous stirring. The mixture was stirred for 4 h at this temperature, and potassium permanganate (15 g) was added to the suspension very slowly. The mixture is diluted with the very slow addition of 200 ml water and kept under stirring for 2 h. Then, the ice bath was removed, and the mixture was stirred at 35 °C for 2 h. The above mixture is kept in a reflux system at 98 °C for 10–15 min. After cooling, the mixture kept under stirring for 2 h at 25 °C. The solution is finally treated with 40 ml of H_2O_2 and then 200 mL of water. Then, it is kept without stirring for 3–4 h, where the particles settle at the bottom and the remaining water is poured through a filter. The resulting mixture is washed repeatedly by centrifugation with 10% HCl and then with deionized (DI) water several times until it forms a gel-like substance (pH-neutral). After centrifugation, the gel-like substance is vacuum dried at 60 °C for more than 6 h to GO powder.

Phosphorylated GO (**PGO**) was obtained through the phosphorylation of graphene oxide using $POCl_3$ as the phosphorus source. The details can be found in the supporting information. In a typical procedure using $POCl_3$ as the phosphorylating agent, 80 mg of lyophilized graphene oxide is dispersed in 250 mL of THF. The mixture is sonicated until the formation of a homogeneous suspension. Then, 42.9 mmol of K_2CO_3 is added to the suspension, and the mixture is kept under stirring for 3 h. Subsequently, 42.9 mmol of $POCl_3$ is dropped into the suspension, and the mixture is kept under stirring for 4 days. The phosphorylated product is dispersed in deionized water and kept

under stirring for 18 h to hydrolyze the residual Cl that did not react with GO. Finally, the product is collected through filtration and is thoroughly washed/rinsed with DI water and ethanol.

Table S1. Contact angle, thermal analysis, and mechanical properties of modified chitosan films.

Sample	Contact Angle	TGA (wt%)[a]			DSC		Mechanical Properties			
		300 °C	500 °C	700 °C	T1 (°C)[b]	T2 (°C)[c]	Tensile Modulus (MPa)	Tensile Strenght (MPa)	Elongation at break	Toughness (MJ/m^3)
**CS-*f***	73.3 ± 2.18	40%	73.5%	100	105	247	1049	35	36%	9.78
**CS-GO-*f***	70.6 ± 1.22	44%	82%	99%	101	296	1926	63	21%	9.90
**CS-PGO-*f***	76.7 ± 1.14	37%	71%	100%	102	295	1677	53	24.5%	10.30
**CS-rGO-*f***	101.1 ± 3.67	36%	75%	97%	97	296	1411	52	31%	12.96
**CS-$SiMe_3$GO-*f***	100.5 ± 2.27	34.5%	57%	62%	97	297	1245	42	33%	10.22

[a] decomposition wt % at a corresponding temperature. [b] endothermic peaks for water evaporation temperature. [c] exothermic peaks of chitosan decomposition.

Table S2. Propidium iodide (PI) permeability (%) of cell membranes of S. *aureus* after incubation with modified chitosan films.

Sample	PI Permeability (%)
Control	4.5 ± 1.94
CS-GO-*f*	81.0 ± 10.79
CS-PGO-*f*	98.48 ± 4.02
CS-$SiMe_3$GO-*f*	96.98 ± 4.87

Solid-state ^{13}C SSNMR of graphene oxide reveals the presence of epoxide groups evidenced by the signal at 60 ppm and the presence of alcohols (signal at 68 ppm), and the signal at 131 ppm is attributed to aromatic carbons of graphitic domains.

Solid-state ^{13}C SSNMR of phosphorylated graphene oxide (PGO) reveals the presence of epoxide groups evidenced by the signal around 63 ppm; the formation of C-O-P bonds might be evidenced by the signal at 69 ppm, and the signal at 127 ppm is attributed to aromatic carbons of graphitic domains.

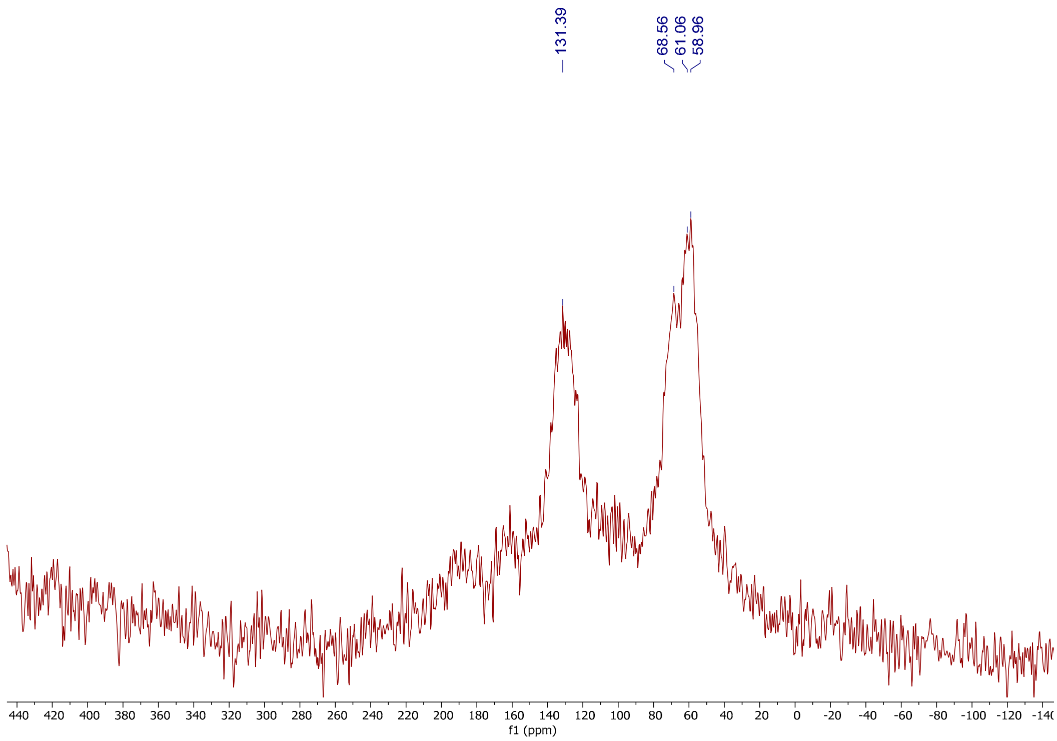

(a)

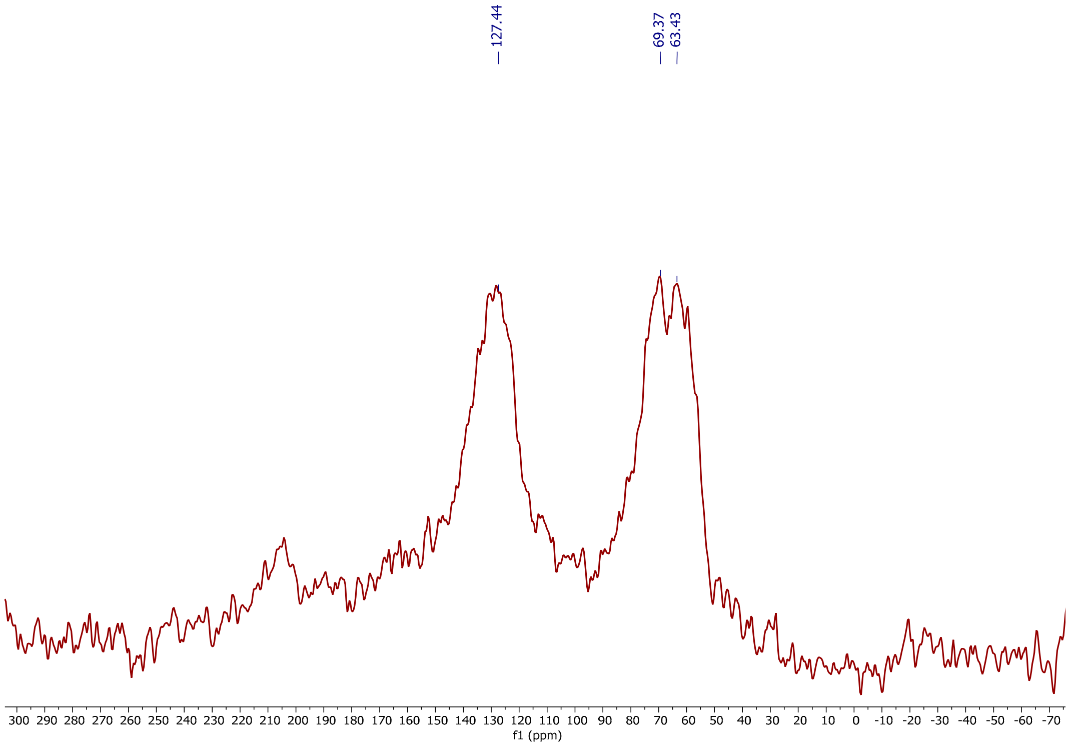

(b)

Figure S1. ^{13}C Solid-State NMR Spectrum of Graphene Oxide. (**a**) ^{13}C Solid State NMR spectrum of graphene oxide; (**b**) ^{13}C Solid-state NMR spectrum of **PGO.**

In GO's Raman spectrum, two bands corresponding to the D band and the G band appear at 1354 cm^{-1} and 1601 cm^{-1}, respectively. The I_D/I_G found for GO is 0.87.

In the case of PGO, these two bands are shifted to lower values (1348 cm^{-1} for the D band and 1586 cm^{-1} for the G band). An increase of the I_D/I_G value is observed (0.91). This increase and the shift of the two bands are a consequence of the creation of more defective sites on the sheets or a decrease of the graphitic domains due to sonication.

In the case of rGO, these two bands are shifted to lower values (1349 cm^{-1} for the D band and 1594 cm^{-1} for the G band). An increase of the I_D/I_G value is observed (1.26).

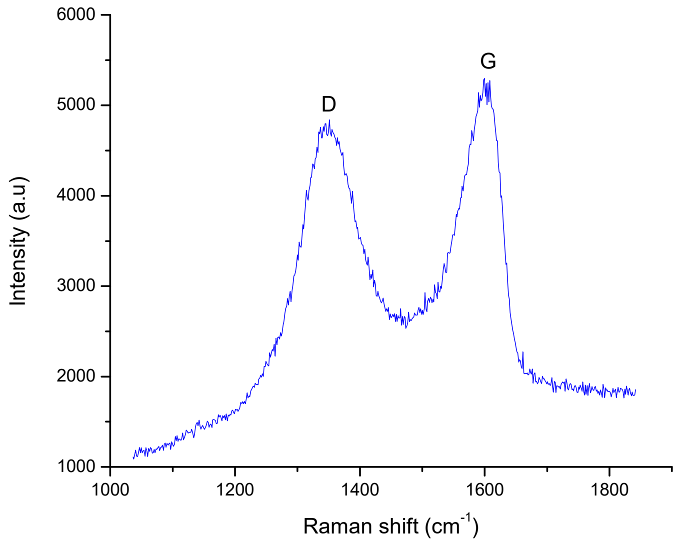

(**a**)

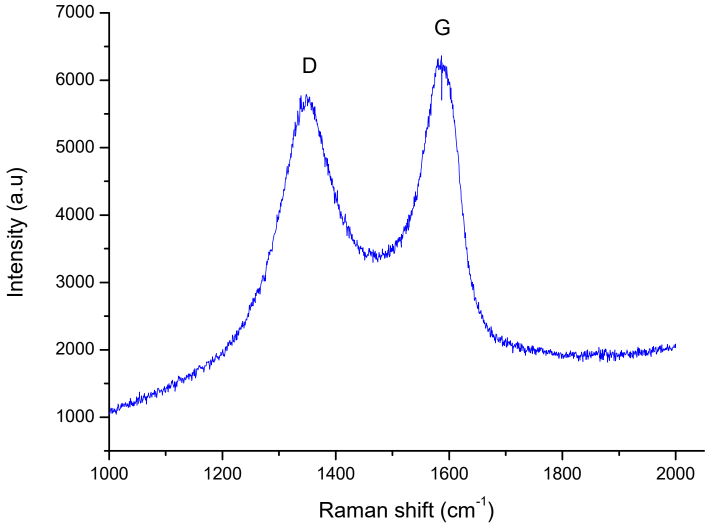

(**b**)

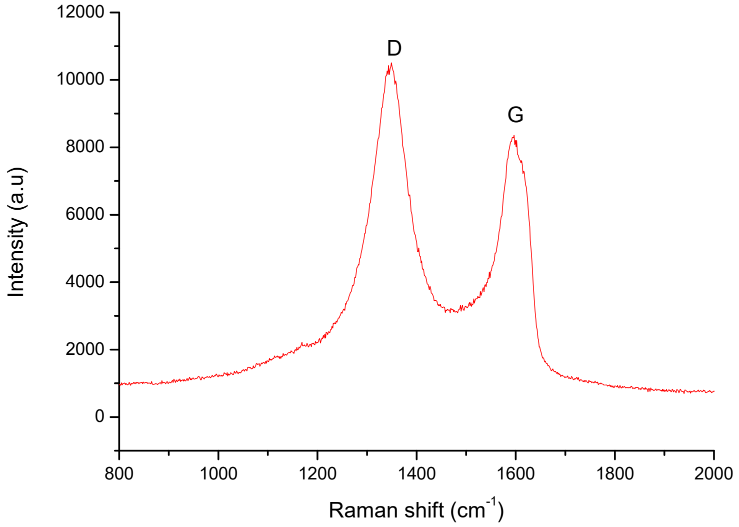

(**c**)

Figure S2. Raman spectrum of **GO** and **PGO.** (**a**) Raman spectrum of **GO;** (**b**) Raman spectrum of **PGO**; (**c**) Raman spectrum of **rGO.**

The integral C1s spectrum of GO can be deconvoluted into three components at 284.5 eV, 286.5 eV, and 288.5 eV corresponding to carbon atoms of graphitic domains, carbon atoms of epoxides and tertiary alcohols, and carboxyl and ester groups carbon O-C=O respectively.

The O1s spectrum of GO evidences the presence of oxygenated functional groups.

The C1s spectrum of PGO is different from the one found for GO. The integral C1s spectrum of PGO can be deconvoluted into four components at 284.5 eV, at 286.3 eV, 287.2 and 288.7 eV, corresponding to carbon atoms of graphitic domains, carbon atoms of tertiary alcohols, carbon atoms of epoxides, and carboxyl and ester groups carbon O-C=O respectively.

The O1s spectrum of GO evidences the presence of oxygenated functional groups.

The P2p spectrum is deconvoluted to one component mainly due to the presence of orthophosphate groups, since the binding energy observed indicates that phosphorus is linked to oxygen atoms.

The integral C1s spectrum of PGO can be deconvoluted into five components at 284.7 eV, 286.2 eV, 286.9, 288.3 eV, and 289.2 eV corresponding to carbon atoms of graphitic domains, carbon atoms of tertiary alcohols, and the carbon atoms of epoxides, carboxyl, and ester groups carbons O-C=O and C-Si, respectively. The 289.2 eV might be due to the subsistence of residual Si–CH_3 groups upon the functionalization of GO with HMDS.

The Si2p spectrum of $SiMe_3GO$ can be deconvoluted to one component, and this is mainly due to the presence of Si–O bonds.

The N1s peak at 401.3 indicates the formation of C-NH and the peak around 399.8 eV suggests the formation of C-N^+ bonds.[2]

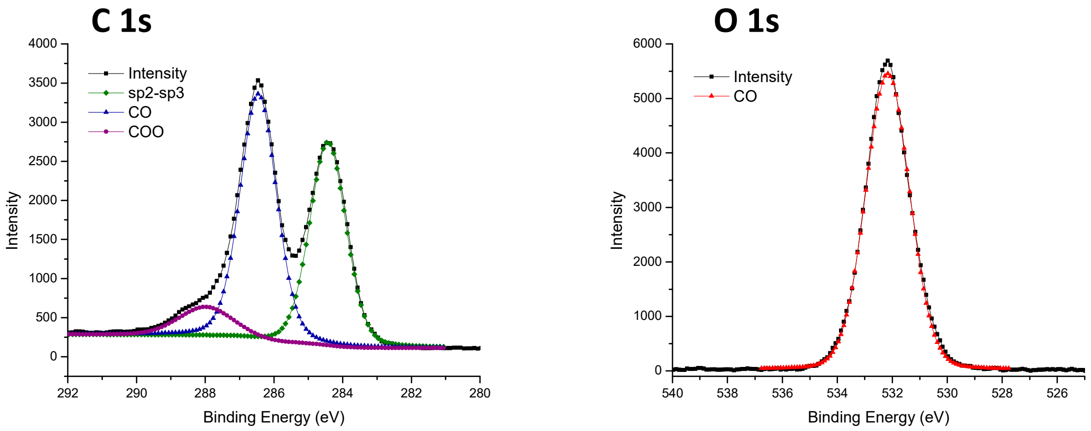

(a)

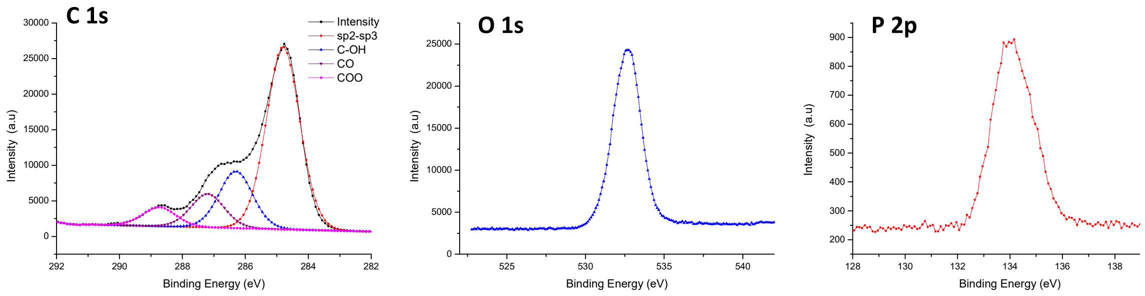

(b)

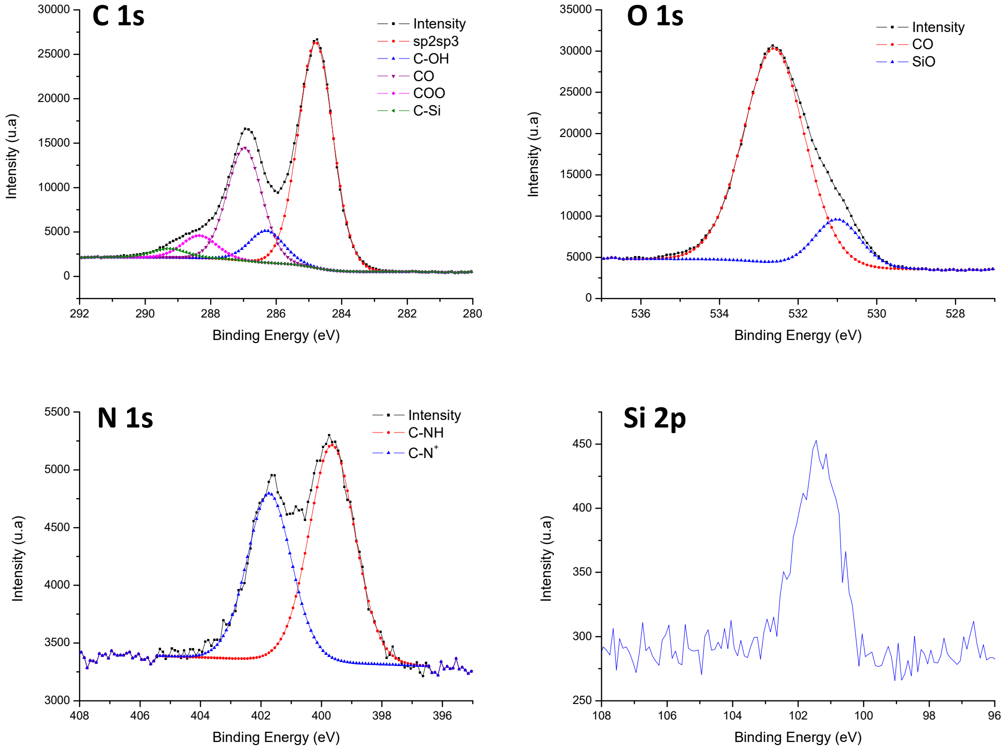

(c)

Figure S3: XPS Analysis of GO, PGO, and SiMe3GO. (**a**) XPS analysis of **GO**; (**b**) XPS analysis of **PGO**; (**c**) XPS analysis of SiMe3GO.

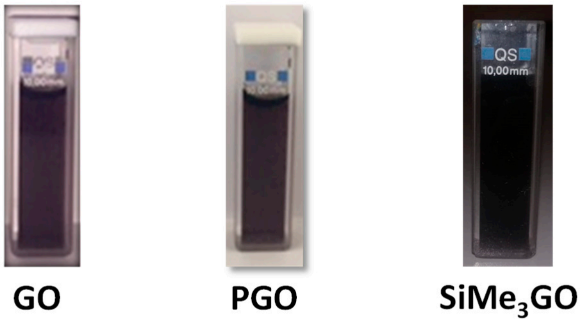

Figure S4. Water dispersion of functionalized graphene fillers.

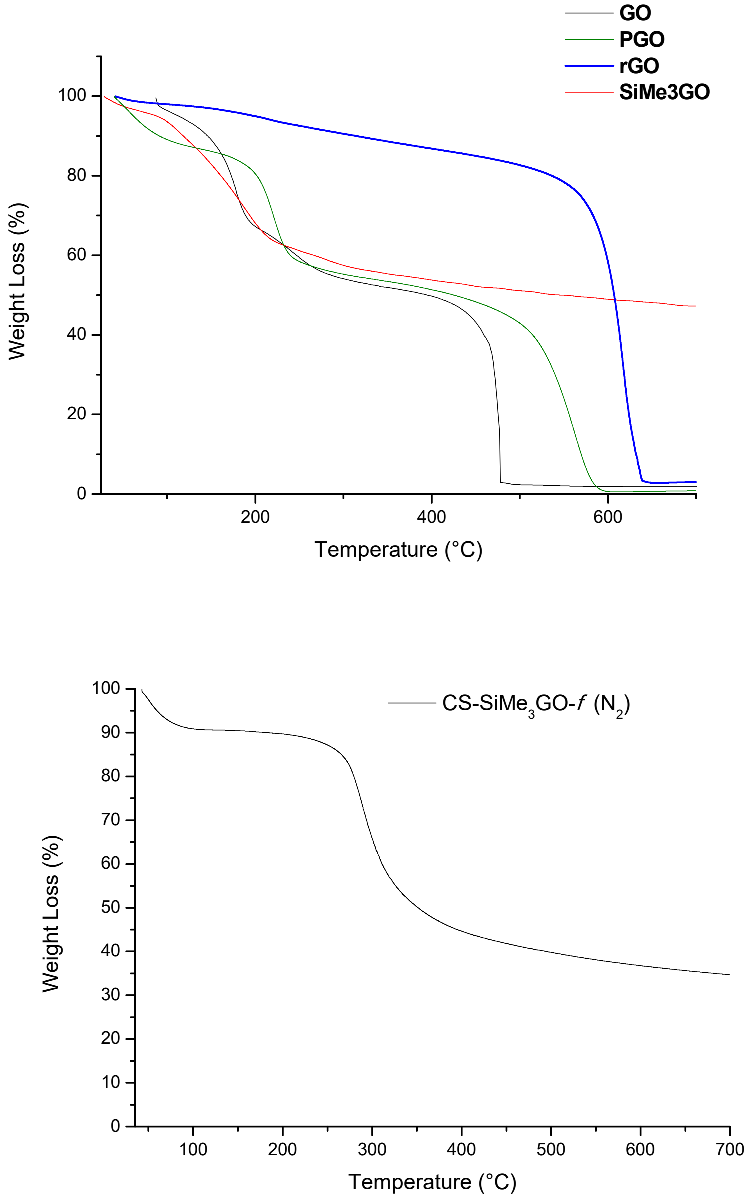

Figure S5. TGA of GO, PGO, rGO, $SiMe_3GO$, and CS-$SiMe_3GO$-*f* (N_2).

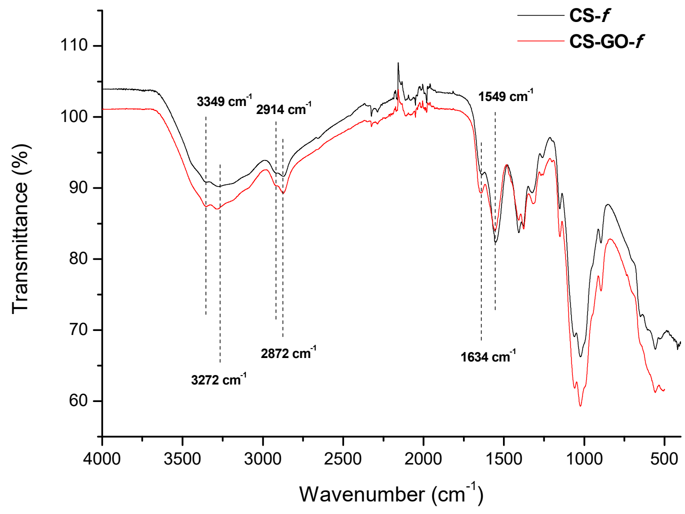
CS-*f*
CS-GO-*f*
3349 cm-1
2914 cm-1
1549 cm-1
2872 cm-1
1634 cm-1
3272 cm-1
Transmittance (%)
Wavenumber (cm-1)
110
100
90
80
70
60
4000
3500
3000
2500
2000
1500
1000
500

(**a**)

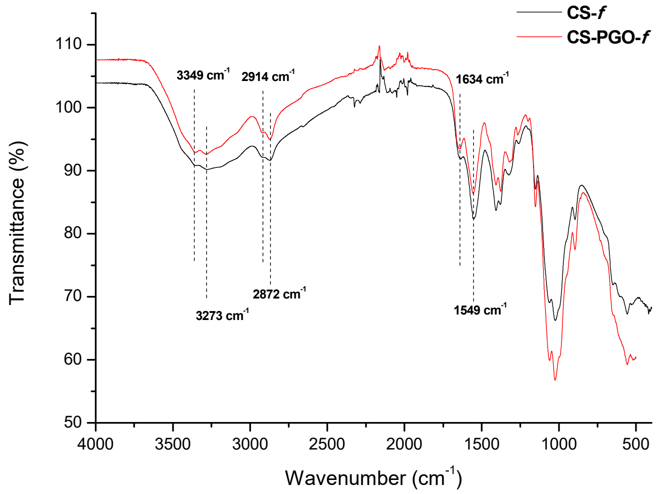
CS-*f*
CS-PGO-*f*
3349 cm-1
2914 cm-1
1634 cm-1
2872 cm-1
3273 cm-1
1549 cm-1
Transmittance (%)
Wavenumber (cm-1)
110
100
90
80
70
60
50
4000
3500
3000
2500
2000
1500
1000
500

(**b**)

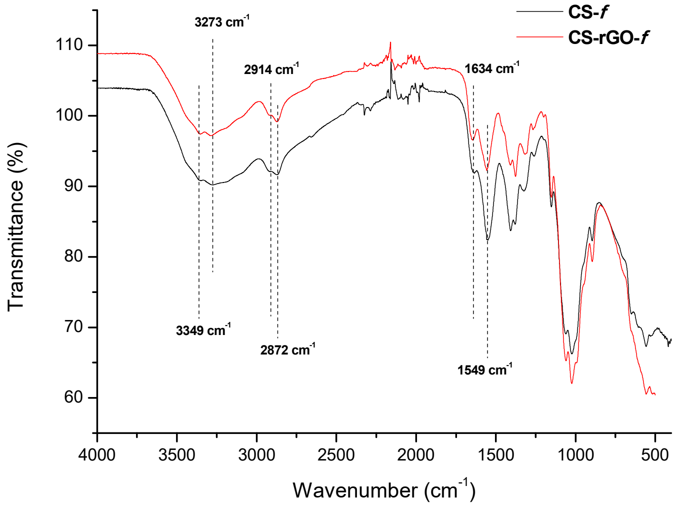
CS-*f*
CS-rGO-*f*
3273 cm-1
2914 cm-1
1634 cm-1
3349 cm-1
2872 cm-1
1549 cm-1
Transmittance (%)
Wavenumber (cm-1)
110
100
90
80
70
60
4000
3500
3000
2500
2000
1500
1000
500

(**c**)

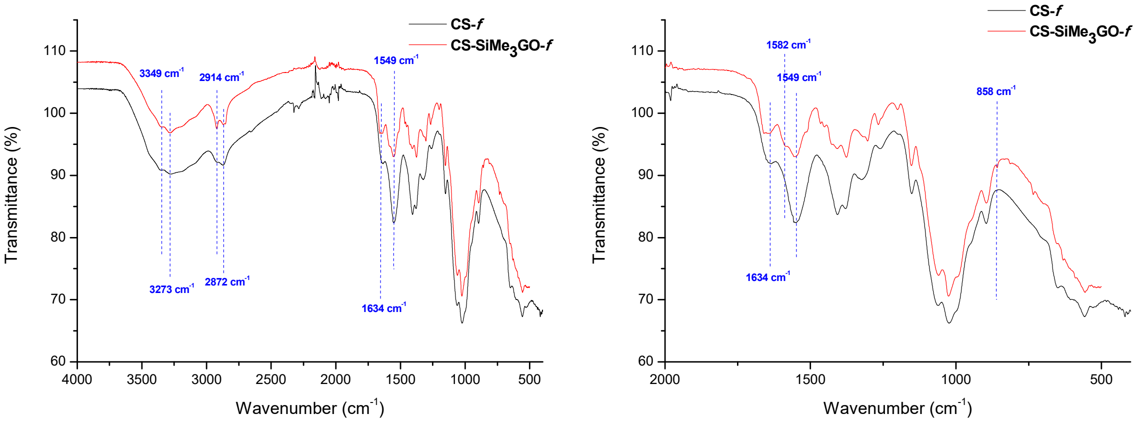

(**d**)

Figure S6. DRIFT analysis of graphene-reinforced chitosan films. (**a**) DRIFT analysis of **CS-GO-*f*;** (**b**) DRIFT analysis of **CS-PGO-*f*;** (**c**) DRIFT analysis of **CS-rGO-*f*;** (**d**) DRIFT analysis of **CS-SiMe$_3$GO-*f***.

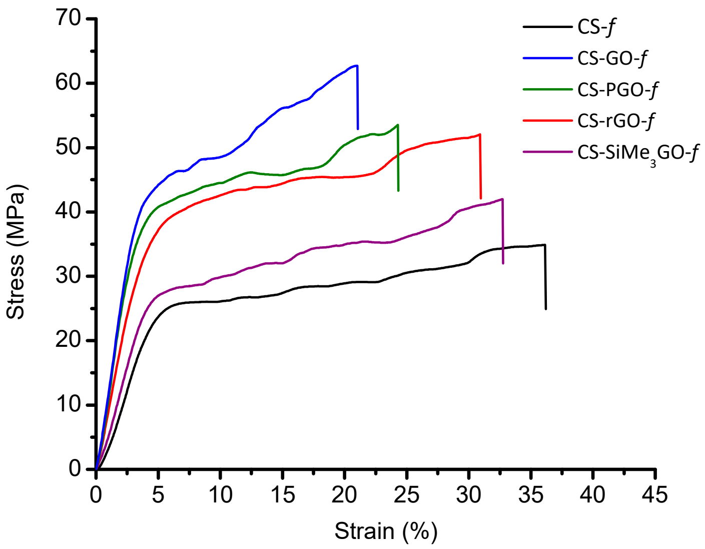

Figure S7. Stress–strain curves of chitosan and chitosan-modified graphene nanocomposites.

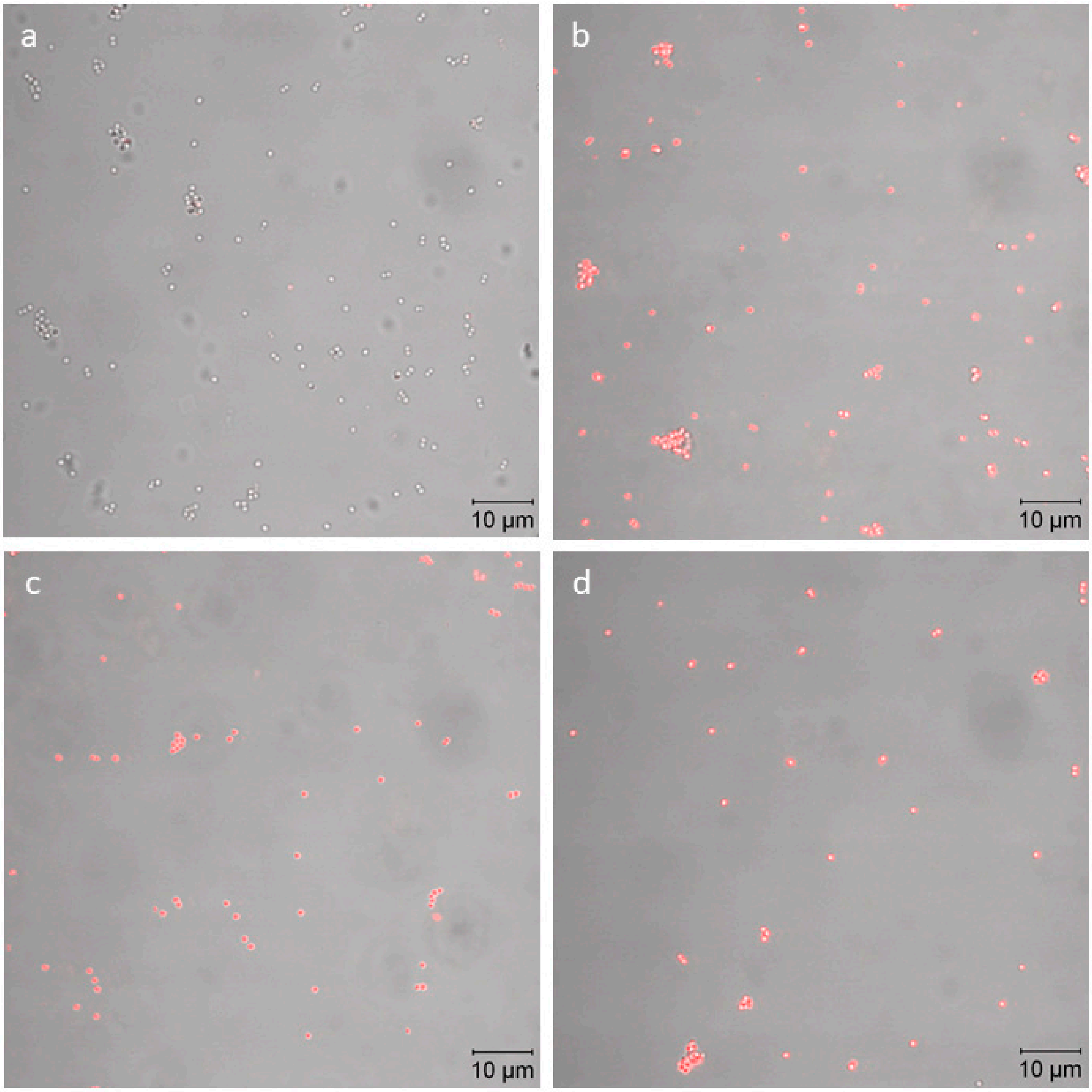

Figure S8. Permeability of *S. aureus* cell membrane after the treatment with chitosan films: (**a**) control, (**b**) **CS-GO-*f***, (**c**) **CS-PGO-*f***, (**d**) **CS-SiMe$_3$GO-*f***.

© 2020 by the authors. Submitted for possible open access publication under the terms and conditions of the Creative Commons Attribution (CC BY) license (http://creativecommons.org/licenses/by/4.0/).
